# What can transactional data reveal about the prevalence of menstrual pain in England?

**DOI:** 10.23889/ijpds.v8i3.2272

**Published:** 2023-09-11

**Authors:** Torty Sivill, Vanja Ljevar, James Goulding, Anya Skatova

**Affiliations:** 1 University of Bristol, The Alan Turing Institute; 2 University of Nottingham; 3 Kubik Intelligence

## Introduction & Background

It has been reported that up to 91% of those who menstruate experience associated pain. Despite its ubiquity, the prevalence of menstrual pain has been under researched due to stigma, disregard from medical professionals and a lack of data. It has also been reported that different demographics experience menstrual pain differently yet the impact of socio-demographic factors on menstrual pain remains to be explored on a national scale due to data scarcity.

## Objectives & Approach

In this study, we propose one way to overcome this data barrier, using a novel measure of menstrual pain extracted from supermarket shopping data. We use these national datasets to identify individual customer behaviour patterns. Specifically, we use transactions involving both a pain and menstrual item as a proxy measure for menstrual pain. We investigate national menstrual pain sales and whether there are significant differences between deprived and less deprived areas of England.

## Relevance to Digital Footprints

This paper brings together data from multiple sources, to provide a population level analysis of the prevalence of menstrual pain England. We use transactional data from a pharmaceutical retailer to develop a novel proxy measure for menstrual pain. We use various machine learning algorithms to explore the relationship between transactional data and various data sources pertaining to social deprivation.

## Results

Our findings indicate that there is a high prevalence of menstrual pain with at least 26.7% of customers who purchase menstrual items also purchasing pain relief simultaneously. These customers are nearly four times more likely to purchase pain relief with a menstrual item than they are without. In addition, our results indicate a significant geographical disparity between menstrual pain transactions. We examine the relationship between a variety of deprivation factors and regional menstrual pain transactions and find average regional income has the highest predictive impact on menstrual pain sales. Contrary to what would expected from previous research, customers from the region with the lowest regional income were a third less likely (32%) to make a menstrual pain transaction than those from the highest income region.

## Conclusions & Implications

This work motivates further research into the national prevalence of menstrual pain to understand why this regional disparity exists and whether it is a consequence of "period poverty''. A better understanding of the sociodemographic factors associated with menstrual pain will help healthcare professionals stratify patients by risk, and could inform strategies to predict and prevent menstrual pain and its adverse impacts.

